# Monitoring the Prestressed Rods in the Basel Border Bridge Maintenance Project: Data Analysis during the Passage of Trucks

**DOI:** 10.3390/s21165570

**Published:** 2021-08-18

**Authors:** Harald Schuler, Florian Meier

**Affiliations:** School of Architecture, Civil Engineering and Geomatics, FHNW University of Applied Sciences and Arts Northwestern Switzerland, Hofackerstrasse 30, 4132 Muttenz, Switzerland; harald.schuler@fhnw.ch

**Keywords:** bridge monitoring, maintenance, fiber optic, corbel, prestressed rods

## Abstract

More than 40 years ago, the expansion joints on the Basel border bridge were constructed using corbels and dapped ends. The consoles had to be reinforced as part of the renovation measures due to damage caused by chloride entry and due to the increased loads. Diagonal rods, which were prestressed, were used. Fiber-optic sensors were additionally installed to these highly stressed rods in order to measure the strains and temperatures. This now makes it possible to measure the actual strains in the strengthening of the corbel, estimate fatigue loads, and set up a warning system in case of overstressing. This article presents the design of the measurement system and the analysis of the data. Furthermore, the reference measurements that can establish the relationship between the measured strains and the loads passed over are presented.

## 1. Introduction

### 1.1. Monitoring

By monitoring infrastructure such as bridges, greater safety for users can be achieved [1,2,3]. This is feasible since progressive damage can be detected at an early stage, safety is ensured, and the repair effort is significantly reduced. In the case of brittle material behavior, even small changes in deformations or strains can indicate damage. Precise and continuous data recording is necessary to detect damage in time. However, this can lead to large amounts of data. Therefore, it makes sense to concentrate on the neuralgic points of the bearing structure and to adjust the sampling rate reasonably.

During the repair of the Basel border bridge, the cantilever benches at the Gerber joints were strengthened by inserting additional prestressed diagonal rods. The monitoring system presented here looks at this neuralgic point of the structure. The fiber-optic strain and temperature sensors were glued onto the rods. These rods were inserted into drilled holes and prestressed. The load changes due to the passage of trucks can now be measured. In case of overstressing, a warning signal is sent and measurements can be obtained. The monitoring project started in 2018 and was set up as a first step that will continue for 10 years until 2028. Further examples of monitoring infrastructures in Switzerland can be found in [4,5] and a general overview in [6].

### 1.2. The Basel Border Bridge

**Figure 1:**
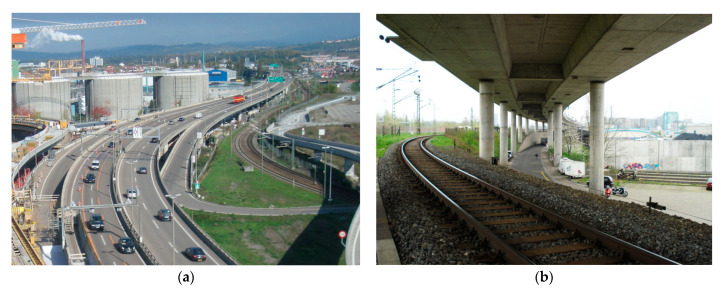
Basel border bridge: (**a**) view from above; (**b**) view from below.

**Figure 2:**
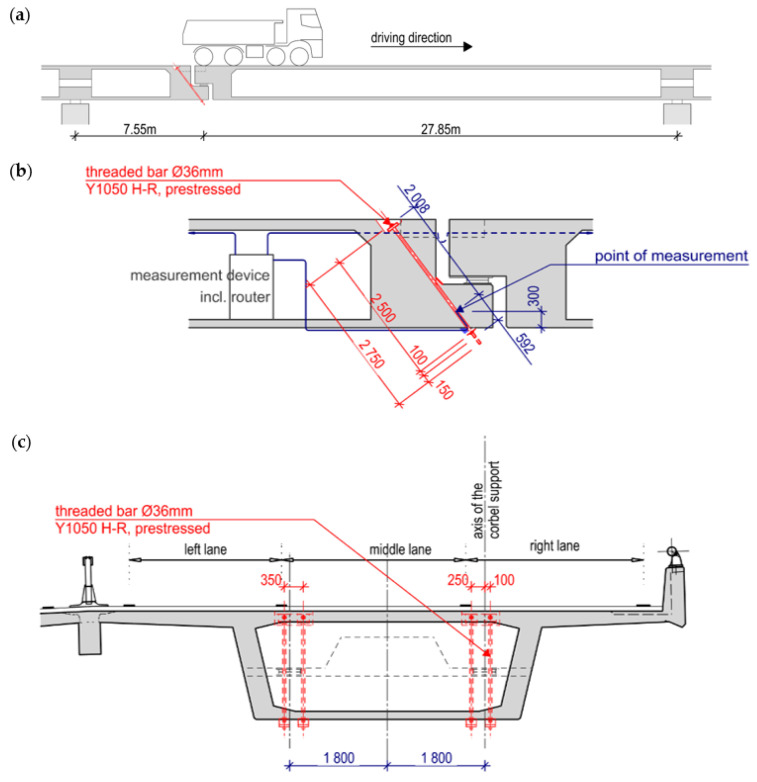
(**a**) One span which includes a Gerber hinge; (**b**) Reinforcement measure with a prestressed threaded rod; (**c**) cross-section of one direction of travel.

Motorway 2 (A2) is one of the busiest north–south traffic axes in Switzerland. It runs from the Italian border at Chiasso through the Gotthard to Basel and connects to the German Autobahn 5 (A5) at the German–Swiss border. In this area, it is routed for a length of 1480 m over the Basel border bridge, of which approx. 950 m is on Swiss territory (Figure 1) and 530 m on German territory. The bridge consists of 42 spans. Figure 2 shows a longitudinal section of one span and the cross-section. The width is about 11 m in one direction of travel. The superstructure has a constant height of two meters and is prestressed in the longitudinal direction and transversely in the crossbeam. In order to be able to absorb the temperature deformations, three dilatation joints (B, C, and D) are arranged. The joints are located in every eleventh span. In between, the spans are built continuously. The length of each span is 35.4 m. The bridge was built between 1976 and 1980. After more than 38 years of operation, a repair was necessary. The dilatation joints were defective and locally showed considerable chloride contamination and reinforcement corrosion. The static recalculation showed clear deficits in the area of the corbels of the Gerber joints. Furthermore, the maintenance measure provided for an adjustment of the lanes and an additional slow lane for trucks in the direction of Germany, which resulted in an increase in the loads. 

### 1.3. Strengthening of the Gerber Joints

The Gerber joints consist of a corbel in the supporting element and a dapped end in the overlying element (Figure 2a,b). In between, there are two bridge supports per box girder. The two supports have a lateral distance of 3.6 m (Figure 2c). In order to increase the insufficient load-bearing capacity of the corbels, two DYWIDAG tension rods (ø36 mm, Y1050) were installed and prestressed at each support. In the transverse direction, the two rods are close to the supports, so that the force on the corbel is transmitted almost directly to the rods. Figure 2 shows the retrofitted tension rods in red and the position where the sensors are placed. During the insertion of the rods into the borehole, extreme caution was necessary to avoid damaging the sensors. The protection measures for the sensors and the prestressing of the rods are described in [7]. 

## 2. Measurement Set-Up

**Figure 3:**
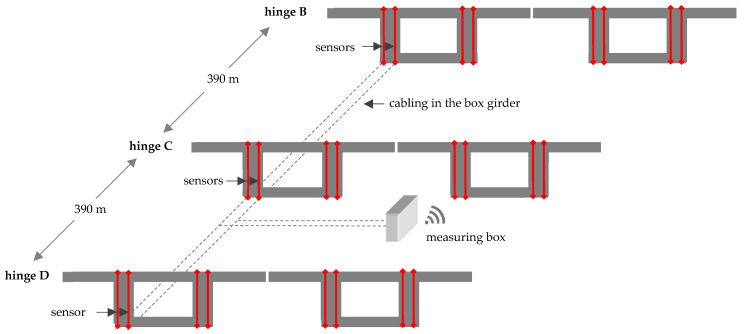
Set-up of the strain and temperature measurement.

**Figure 4:**
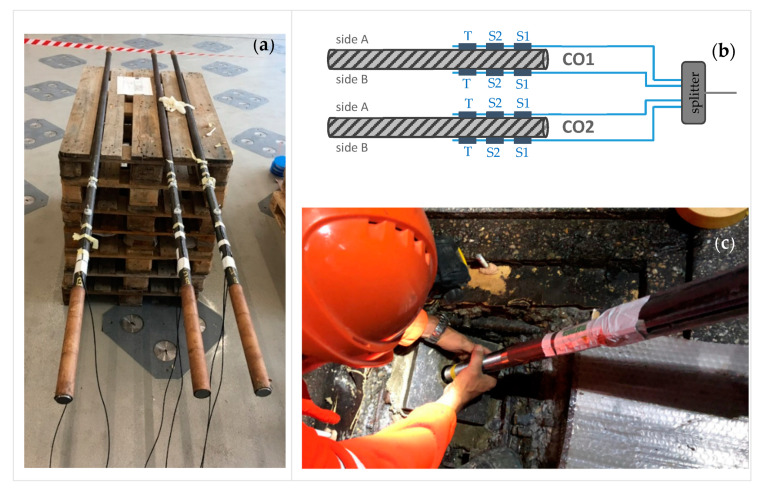
(**a**) Assembling of the optical sensors in the laboratory; (**b**) location of the strain (S) and temperature (T) sensors at each Gerber hinge, here, hinge C; (**c**) inserting a rod into the borehole.

In this section, the measurement set-up along the Basel border bridge is explained. As mentioned in the previous section, the Gerber joints with a corbel and a dapped end are located in every eleventh span. The distance between these expansion joints is 390 m. As Figure 3 shows, only two of eight rods are instrumented at each expansion joint. The sensors are located on the side where the slow lane for the trucks is planned. The bars on this side are the most stressed. The fiber-optic cables are laid in the girder box along the longitudinal direction and merge in the measuring box at the Gerber joint C. The fiber optical measurement system was installed by Marmota Engineering AG. A HYPERION si155 from Luna Innovations (former Micron Optics) was used as interrogator, combined with (os1100) strain and (os4300) temperature sensors also from Luna Inc. The theoretical accuracy of the installed Systems is about 1 με resp. 0.1 °C. The thermo-optic compensation as well as the referencing was applied according to state-of-the-art and best practice. Further information on fiber optical measurements can be found in [8,9]. In total, six rods are instrumented with four strain sensors, and two temperature sensors each. Two of each are arranged opposite each other. This means that bending stresses can also be estimated. The deviation of the strains between sides A and B was less than 20%. Figure 4a,b shows the application and position of the strain and temperature sensors. Not all sensors provide plausible results. About 10% failed and 30% produced unrealistic results. The reason could be damage to the sensor or the adhesive during insertion of the rod into the borehole (Figure 4c) or during injection of the grout.

## 3. Data Acquisition

### 3.1. Recorded Data

The data recording has two main objectives: long-term monitoring of the bridge and short-term monitoring to record peak loads caused by the passage of heavy loads.

#### 3.1.1. Long-Term Monitoring

The recorded data should help to detect long-term changes in the load-bearing capacity of the corbel. Over a period of several years or decades, the strains in the rods are monitored. If an increase is observed, a weakening of the corbel is probable. For example, if corrosion in the existing reinforcement increases, the stresses are redistributed in the corbel and more load is carried by the instrumented rods. Such a change in the strains is measurable and indicates damage in the corbel.

#### 3.1.2. Short-Term Monitoring

**Figure 5:**
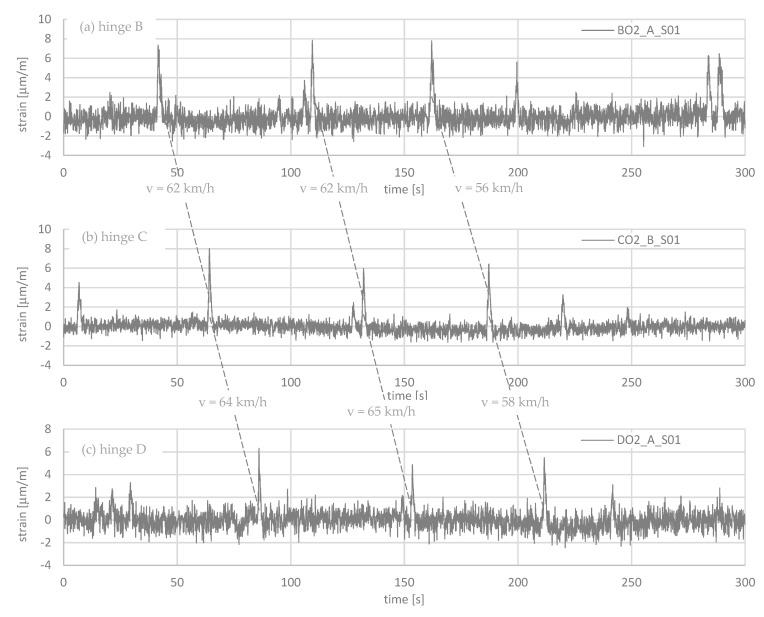
Strain measurement over a period of 5 min with a sampling rate of 1/10 s: (**a**) Gerber hinge B; (**b**) Gerber hinge C; (**c**) Gerber hinge D. The distance between the Gerber hinges is 390 m.

Short-term monitoring focuses on the stresses in the rods due to the passage of heavy loads. Trucks with a total mass of more than 10 tons can be recorded. It is not intended to measure the passage of cars, as their mass is too low. The aim is to evaluate the stresses in the rods in selected time periods, e.g., hourly, daily, weekly, or in rush hours. The stresses in the rods can be accumulated, and actual fatigue loads can be compared to designed loads. Figure 5 shows the recording of the strains at joints B, C, and D over a period of 5 min (300 s). The distances between the Gerber joints are 390 m. Single trucks can be captured, as the three consecutive figures show. Even the velocity of the trucks can be determined. The mass of the crossing trucks is unknown. The load-bearing is complex, including existing reinforcement and new prestressed rods. However, different amplitudes can be seen clearly, which certainly results from the different masses of the trucks. Hence, in Section 4, reference measurements are reported when defined loaded trucks were driven over the bridge.

### 3.2. Sampling Rate

In order to reduce the amount of data to be stored, the effect of the sampling rate on the signals was studied. The goal was to find a sufficient sampling rate to capture the course and amplitude of the signal precisely when a truck is passing. In total, 36 sensors were installed on six rods at three Gerber joints: four strain and two temperature gages on each rod. The data were written to text files and stored every hour. Table 1 shows the amount of data depending on the sampling rate for a period of one hour and one year. As can be seen, for a sampling rate of 0.01 s, 2.3 TB must be stored for the signals from 36 sensors over a period of one year. This is not feasible for this project, which is planned for a period of several decades. To keep the data management simple, a reasonable sampling rate should be found.

**Figure 6:**
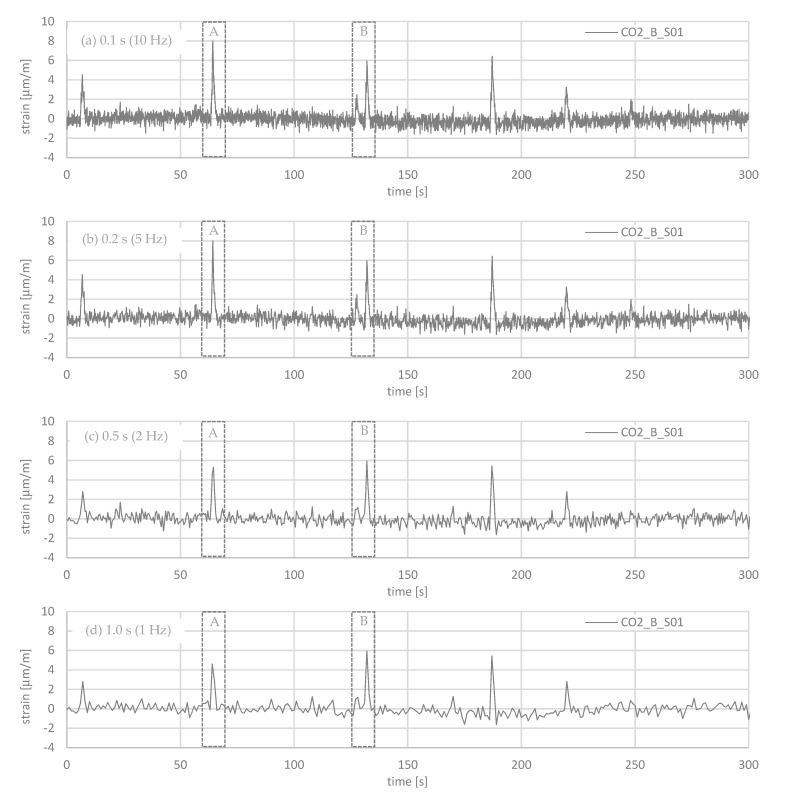
Influence of the sampling rate to the strain measurement over a period of 5 min at Gerber hinge C: (**a**) 0.1 s (10 Hz); (**b**) 0.2 s (5 Hz); (**c**) 0.5 s (2 Hz); (**d**) 1.0 s (1 Hz).

The influence of the sampling rate to the recorded signals was studied at Gerber joint C. Four sampling rates were considered: 0.1 s (10 Hz), 0.2 s (5 Hz), 0.5 s (2 Hz), and 1.0 s (1 Hz). As Figure 6a,b shows, a sampling rate of 0.2 s can reproduce the amplitude of the signals almost as well as a sampling rate of 0.1 s. As Figure 6c,d shows, for sampling rates of 0.5 and 1.0 s, the amplitudes are reduced significantly. Hence, a precise measurement of the strains is not possible with these sampling rates. To study this more precisely, a shorter period is considered. 

**Figure 7:**
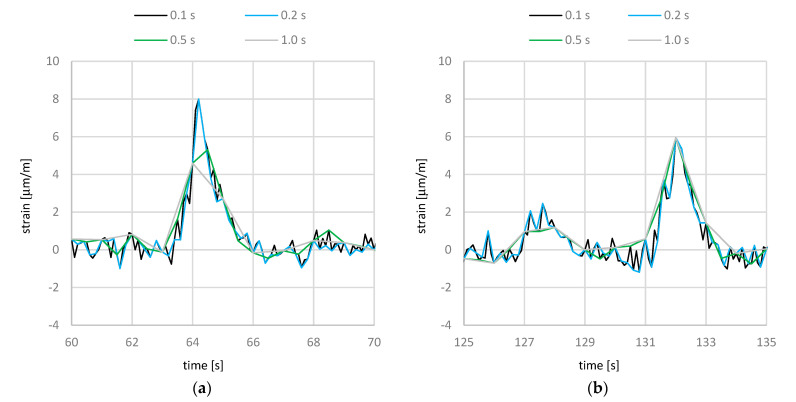
Influence of the sampling rate to the strain measurement over a duration of 10 s at Gerber hinge C: (**a**) time period A from Figure 6; (**b**) time period B from Figure 6.

Considering a duration of 10 s (Figure 7), the influence of the sampling rate on the recording could be analyzed precisely. As expected from the previous figure, the difference between a sampling rate of 0.1 and 0.2 s is small (cp. Figure 7). For 0.1 s, the oscillations are more pronounced, which results, however, mainly from the measurement. The measurements with sampling rates of 0.5 and 1.0 s are not precise enough to capture the course and the amplitude of the strain measurements in the rods. Compared to the sampling rate with 0.1 s, the amplitude is less than 70% for 0.5 s and less than 60% for 1.0 s. With a length from the Gerber joint to the next support (27.85 m) and an estimated velocity (16.7 m/s = 60 km/h cp; Figure 5), the load duration for a passing truck can be calculated roughly: t = 27.85 m/16.7 m/s = 1.7 s. This corresponds quite well to the measured duration in Figure 7. As can be seen, for lower sampling rates of 0.5 and 1.0 s, the course flattens, and the duration becomes larger. Hence, to capture the duration precisely, the recording must be obtained every 0.2 s or more often.

## 4. Reference Measurements with Defined Loaded Trucks

**Figure 8:**
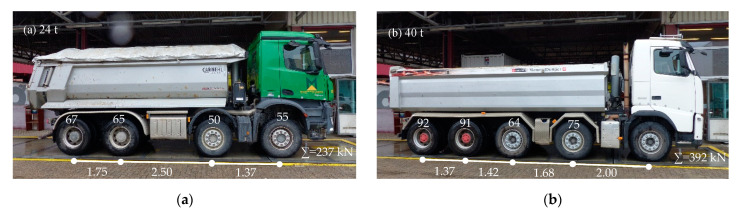
Axle loads (kN) and axle distances (m) of the trucks in the reference measurements: (**a**) truck with a total mass of 24 t; (**b**) truck with a total mass of 40 t.

**Figure 9:**
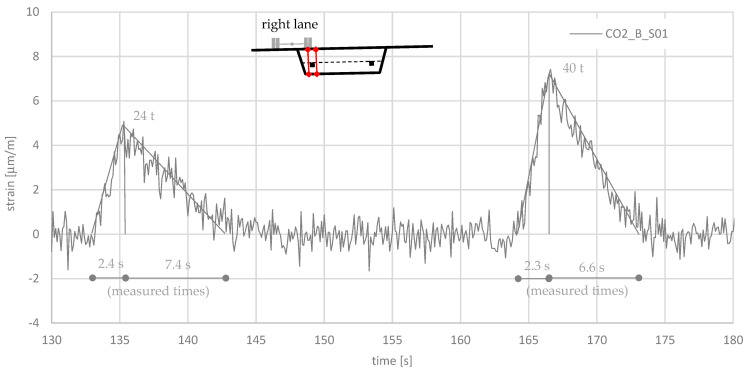
Strain measurement of the reference trucks, where after the 24 ton truck, the 40 ton truck followed.

The load introduction from a passing truck into the prestressed steel rods is complicated and almost impossible to recalculate. A simple approach with a truss model was tried in [7]. In order to obtain a correlation between passing truckloads and the measured strains, reference measurements were conducted. Therefore, two trucks with defined loads were driven over the bridge. The axle loads and axle distances were measured and are shown in Figure 8. The total mass of each trucks was 40 and 24 tons. The trucks were driven over the bridge with a velocity of about 12 km/h. Simultaneously, the strain was measured at the Gerber hinges. Figure 9 shows the measured strains at Gerber hinge C, where the 24 ton truck drove first and the 40 ton truck drove second. The difference in the strain measurements is clearly visible, although the amplitudes are very small (≤10 μm/m). The ratio of the strain amplitudes, 5/7.5 μm/m = 0.67, is almost equal to the ratio of the loads, 24/40 t = 0.6, which motivated us to use a linear relationship between loads and measured strains. We attempted to recalculate the shape of the measured signal. For the 24 ton truck with a length of 5.62 m, the rise time was 5.62 m/((12/3.6) m/s) = 1.7 s. For the 40 ton truck, the rise time was 6.47 m/((12/3.6) m/s) = 1.9 s. As Figure 9 shows, the measured rise times were slightly greater than the calculated one. For the descending branch, the calculated duration was for the 24 ton truck (27.85 m−5.62 m/2)/((12/3.6) m/s) = 7.5 s. For the 40 ton truck, the duration was (27.85 m−6.47 m/2)/(12/3.6 m/s) = 7.4 s. In summary, the measurement and calculation agreed quite well with each other (see Figure 9). The plausibility of the measurements can be confirmed by a simple calculation.

A comparison of the amplitude in normal operation in Figure 7a with the amplitude of the 40 ton truck in the reference measurements in Figure 9 shows a higher value of about 7% (8 μm/m/7.5 μm/m = 1.07). Assuming that the permissible total mass of 40 tons was not exceeded in Figure 7a, a dynamic increase can be assumed. The difference in speed between normal operation and the reference measurements was 60 to 12 km/h. The observation of further amplitudes in normal operation, which are not shown in this publication, confirm a dynamic increase for higher speeds.

In order to conduct further data analysis, e.g., to accumulate different stress amplitudes for the calculation of “real” fatigue loads, a correlation between the measured strain and the passing truck load is required. A simple approach for the allocation of the measured strains to the truck loads could be:
• 6 μm/m ≤ ε_max_=>30 t ≤ truck mass• 4 μm/m ≤ ε_max_ < 6 μm/m=>20 t ≤ truck mass < 30 t• 2 μm/m ≤ ε_max_ < 4 μm/m=>10 t ≤ truck mass < 20 t

This rule applies to the Gerber joint C and for trucks driving in the right lane (see Figure 2c). For trucks in the middle lane, the amplitude is about half as large, and in the left lane, no deflection is expected when a truck passes the measuring point.

## 5. Discussion

As part of the A2 Basel maintenance project, the Gerber joints were strengthened to resist the increased loads, e.g., from the truck slow lane before the German border. Each box girder of the bridge has two supports, which were strengthened with two DYWIDAG tension rods (ø36 mm, Y1050) each, at joints B, C, and D. Near the slow lane, the rods are instrumented with four optical strain sensors and two optical temperature sensors. The measured data are stored onsite and transmitted via the mobile phone network to data servers at the FHNW University of Applied Sciences and Arts Northwestern Switzerland. The following findings were obtained from the monitoring project:

**Measurement set-up**

• The optical sensors on the rods must be very well-protected to avoid damage during inserting into the borehole, post-tensioning, and grouting. Although the sensors were protected through spacers and were well-encased, some sensors failed.
• Data acquisition and control of the onsite computers are possible via a mobile network. However, disturbances, such as an interruption in the power supply or other interventions, cannot be ruled out completely. Hence, an easy access to the measuring box is indispensable in order to rectify minor faults quickly.

**Data acquisition**

• The passage of trucks can be traced at every Gerber joint, although the measured strains are very small, less than 10 μm/m. The amplitudes at the Gerber joints B, C, and D differ ±20% for the same truck, related to the mean value of the maximum strains. Reasons for the differences may be: a different amount of overcompressed concrete, which results in different stiffnesses of the prestressed tension chords, or flexure in the rods, which can result from a non-straight borehole or from a canting of the anchor plate at the borehole entrance. Nevertheless, trucks with different masses can be measured quite well, and they can be followed when they cross the hinges B, C, and D.
• With the help of a study on the sampling rate, the correct sampling rate for the given bridge was found. The span of the bridge is 35.4 m and the Gerber joints are located at a distance of 7.55 m from the closer supports. With a sampling rate of 0.2 s (5 Hz) or less, the passage of the trucks can be recorded sufficiently accurately. With sampling rates of 0.5 s (2 Hz) and 1.0 s (1 Hz), the amplitudes are up to 40% too small. If sampling is more frequent than every 0.2 s (5 Hz), the amount of data increases significantly, almost linearly with the storage frequency.

**Reference measurements**

• The reference measurements with defined loaded trucks showed that the difference between 24 and 40 ton trucks is clearly measurable. A simple correlation between the measured strains and the masses of the trucks could be established in 10 ton increments. This applies to the passaging of the bridge, in the slow lane in the direction of travel on the right-hand side. In the left lane, the strains should be very small, as the entire load flows into the left support. Furthermore, the rise and fall times agree relatively well between the measurement and the calculated estimates.
• The dynamic factor was not evaluated within the strain measurements. However, the measured strains in normal operation, at a maximum speed of 60 km/h, are up to 10% higher than in the reference measurements at a speed of 12 km/h. This indicates that a dynamic increase occurs due to the roadway transition at the Gerber joints. However, the amplitudes of the strains are too small for a quantitative statement on the dynamic factor.

In the next step, the automatic analysis of data is planned (hourly, daily, weekly, etc.). Amplitudes and possibly other variables are to be statistically analyzed. The aim is to determine actual fatigue loads. The system will be used as extensively as possible to measure long- and short-term effects. The warning system is already installed and can be read about in [7].

## Figures and Tables

**Table 1:** Required storage space for the data from 36 sensors for a period of 1 h and 1 year depending on the sampling rate.

	0.01 s (100 Hz)	0.1 s (10 Hz)	1.0 s (1 Hz)
1 h	263 MB	26.3 MB	2.6 MB
1 year	2304 GB	230 GB	23 GB
